# Childhood obesity, prevalence and prevention

**DOI:** 10.1186/1475-2891-4-24

**Published:** 2005-09-02

**Authors:** Mahshid Dehghan, Noori Akhtar-Danesh, Anwar T Merchant

**Affiliations:** 1 Population Health Research Institute, McMaster University, Hamilton, Canada; 2 School of Nursing and Department of Clinical Epidemiology and Biostatistics, McMaster University, Hamilton, Canada; 3 Department of Clinical Epidemiology and Biostatistics, and Population Health Research Institute, McMaster University, Hamilton, Canada

## Abstract

Childhood obesity has reached epidemic levels in developed countries. Twenty five percent of children in the US are overweight and 11% are obese. Overweight and obesity in childhood are known to have significant impact on both physical and psychological health. The mechanism of obesity development is not fully understood and it is believed to be a disorder with multiple causes. Environmental factors, lifestyle preferences, and cultural environment play pivotal roles in the rising prevalence of obesity worldwide. In general, overweight and obesity are assumed to be the results of an increase in caloric and fat intake. On the other hand, there are supporting evidence that excessive sugar intake by soft drink, increased portion size, and steady decline in physical activity have been playing major roles in the rising rates of obesity all around the world. Consequently, both over-consumption of calories and reduced physical activity are involved in childhood obesity.

Almost all researchers agree that prevention could be the key strategy for controlling the current epidemic of obesity. Prevention may include primary prevention of overweight or obesity, secondary prevention or prevention of weight regains following weight loss, and avoidance of more weight increase in obese persons unable to lose weight. Until now, most approaches have focused on changing the behaviour of individuals in diet and exercise. It seems, however, that these strategies have had little impact on the growing increase of the obesity epidemic. While about 50% of the adults are overweight and obese in many countries, it is difficult to reduce excessive weight once it becomes established. Children should therefore be considered the priority population for intervention strategies. Prevention may be achieved through a variety of interventions targeting built environment, physical activity, and diet. Some of these potential strategies for intervention in children can be implemented by targeting preschool institutions, schools or after-school care services as natural setting for influencing the diet and physical activity. All in all, there is an urgent need to initiate prevention and treatment of obesity in children.

## Introduction

Childhood obesity has reached epidemic levels in developed countries. Twenty five percent of children in the US are overweight and 11% are obese. About 70% of obese adolescents grow up to become obese adults [1-3]. The prevalence of childhood obesity is in increasing since 1971 in developed countries (Table 1). In some European countries such as the Scandinavian countries the prevalence of childhood obesity is lower as compared with Mediterranean countries, nonetheless, the proportion of obese children is rising in both cases [4]. The highest prevalence rates of childhood obesity have been observed in developed countries, however, its prevalence is increasing in developing countries as well. The prevalence of childhood obesity is high in the Middle East, Central and Eastern Europe [5]. For instance, in 1998, The World Health Organization project monitoring of cardiovascular diseases (MONICA) reported Iran as one of the seven countries with the highest prevalence of childhood obesity. The prevalence of BMI (in percentage) between 85th and 95th percentile in girls was significantly higher than that in boys (10.7, SD = 1.1 vs. 7.4, SD = 0.9). The same pattern was seen for the prevalence of BMI > 95th percentile (2.9, SD = 0.1 vs. 1.9, SD = 0.1) [6]. In Saudi Arabia, one in every six children aged 6 to 18 years old is obese [7]. Furthermore, in both developed and developing countries there are proportionately more girls overweight than boys, particularly among adolescent [6,8,9].

**Table 1:** Changes in the prevalence of overweight and obesity in some developed countries

**Country/Year**	**Age/yr**	**Study (author)**	**Change in obesity**
**USA**			
1973–1994	5–24	Bogalusa [67]	Mean level increased 0.2 kg/yr, twofold increase in prevalence of obesity
1971–1974	6–19	NHANES I [68]	Relatively stable
1976–1980	6–19	NHANES II [68]	Relatively stable
1988–1994	6–19	NHANES III [68]	Doubled to 11%
1999–2000	6–19	NHANES IV [68]	Increased by 4%
**Japan**			
1974–1993	6–14	Kotani [69]	Doubled (5% to 10%)
**UK**			
1984–98	7–11	Lobstein [70]	Changed from 8% to 20%
**Spain**			
1985/6 to 1995/6	6–7	Moreno [71]	Changed from 23% to 35%
**France**			
1992–1996	5–12	Rolland-Cachera [72]	Changed from 10% to 14%
**Greece**			
1984–2000	6–12	Krassas [73]	Increased by 7%

Overweight and obesity in childhood have significant impact on both physical and psychological health; for example, overweight and obesity are associated with Hyperlipidaemia, hypertension, abnormal glucose tolerance, and infertility. In addition, psychological disorders such as depression occur with increased frequency in obese children [10]. Overweight children followed up for 40 [11] and 55 years [12] were more likely to have cardiovascular and digestive diseases, and die from any cause as compared with those who were lean.

## Definition of childhood obesity

Although definition of obesity and overweight has changed over time [13,14], it can be defined as an excess of Body Fat (BF). There is no consensus on a cutoff point for excess fatness of overweight or obesity in children and adolescents. Williams et al. [15] measured skin fold thickness of 3320 children aged 5–18 years and classified children as fat if their percentage of body fat was at least 25% and 30%, respectively, for males and females. The Center for Disease Control and Prevention defined overweight as at or above the 95^th ^percentile of BMI for age and "at risk for overweight" as between 85^th ^to 95^th ^percentile of BMI for age [16,17]. European researchers classified overweight as at or above 85th percentile and obesity as at or above 95th percentile of BMI [18].

There are also several methods to measure the percentage of body fat. In research, techniques include underwater weighing (densitometry), multi-frequency bioelectrical impedance analysis (BIA) and magnetic resonance imaging (MRI). In the clinical environment, techniques such as body mass index (BMI), waist circumference, and skin fold thickness have been used extensively. Although, these methods are less accurate than research methods, they are satisfactory to identify risk. While BMI seems appropriate for differentiating adults, it may not be as useful in children because of their changing body shape as they progress through normal growth. In addition, BMI fails to distinguish between fat and fat-free mass (muscle and bone) and may exaggerate obesity in large muscular children. Furthermore, maturation pattern differs between genders and different ethnic groups. Studies that used BMI to identify overweight and obese children based on percentage of body fat have found high specificity (95–100%), but low sensitivity (36–66%) for this system of classification [19]. While health consequences of obesity are related to excess fatness, the ideal method of classification should be based on direct measurement of fatness. Although methods such as densitometry can be used in research practice, they are not feasible for clinical settings. For large population-based studies and clinical situations, bioelectrical impedance analysis (BIA) is widely used. Cross-sectional studies have shown that BIA predicts total body water (TBW), fat-free mass (FFM), and fat mass or percentage of body fat (%BF) among children [20-23]. Also, it has been shown that BIA provides accurate estimation of changes on %BF and FFM over time [24]. Waist circumference, as a surrogate marker of visceral obesity, has been added to refine the measure of obesity related risks [25]. Waist circumference seems to be more accurate for children because it targets central obesity, which is a risk factor for type II diabetes and coronary heart disease. To the best of our knowledge there is no publication on specific cut off points for waist circumference, but there are some ongoing studies.

## Causes of obesity

Although the mechanism of obesity development is not fully understood, it is confirmed that obesity occurs when energy intake exceeds energy expenditure. There are multiple etiologies for this imbalance, hence, and the rising prevalence of obesity cannot be addressed by a single etiology. Genetic factors influence the susceptibility of a given child to an obesity-conducive environment. However, environmental factors, lifestyle preferences, and cultural environment seem to play major roles in the rising prevalence of obesity worldwide [26-29]. In a small number of cases, childhood obesity is due to genes such as leptin deficiency or medical causes such as hypothyroidism and growth hormone deficiency or side effects due to drugs (e.g. – steroids) [30]. Most of the time, however, personal lifestyle choices and cultural environment significantly influence obesity.

## Behavioral and social factors

### I. Diet

Over the last decades, food has become more affordable to larger numbers of people as the price of food has decreased substantially relative to income and the concept of 'food' has changed from a means of nourishment to a marker of lifestyle and a source of pleasure. Clearly, increases in physical activity are not likely to offset an energy rich, poor nutritive diet. It takes between 1–2 hours of extremely vigorous activity to counteract a single large-sized (i.e., >=785 kcal) children's meal at a fast food restaurant. Frequent consumption of such a diet can hardly be counteracted by the average child or adult [31].

#### Calorie intake

although overweight and obesity are mostly assumed to be results of increase in caloric intake, there is not enough supporting evidence for such phenomenon. Food frequency methods measure usual diet, but estimate caloric intake poorly [32]. Other methods such as 24-hour recall or food diaries evaluate caloric intakes more accurately, however, estimate short-term not long-term intake [32]. Total energy intake is difficult to measure accurately at a population level. However, a small caloric imbalance (within the margin of error of estimation methods) is sufficient over a long period of time to lead to obesity. With concurrent rise in childhood obesity prevalence in the USA, the National Health and Nutrition Examination Survey (NHANES) noted only subtle change in calorie intake among US children from the 1970s to 1988–1994. For this period, NHANES III found an increase calorie intake only among white and black adolescent females. The same pattern was observed by the latest NHANES (1999–2000). The Bogalusa study which has been following the health and nutrition of children since 1973 in Bogalusa (Louisiana), reported that total calorie intake of 10-year old children remained unchanged during 1973–1988 and a slight but significant decrease was observed when energy intake was expressed per kilogram body weight [33]. The result of a survey carried out during the past few decades in the UK suggested that average energy intakes, for all age groups, are lower than they used to be [34]. Some small studies also found similar energy intake among obese children and their lean counterparts [6,35-37].

#### Fat intake

while for many years it has been claimed that the increase in pediatric obesity has happened because of an increase in high fat intake, contradictory results have been obtained by cross-sectional and longitudinal studies. Result of NHANES has shown that fat consumption of American children has fallen over the last three decades. For instance; mean dietary fat consumption in males aged 12–19 years fell from 37.0% (SD = 0.29%) of total caloric intake in 1971–1974 to 32.0% (SD = 0.42%) in 1999–2000. The pattern was the same for females, whose fat consumption fell from 36.7% (SD = 0.27%) to 32.1% (SD = 0.61%) [38,39]. Gregory et al. [40] reported that the average fat intake of children aged 4–18 years in the UK is close to the government recommendation of 35% energy. On the other hand, some cross-sectional studies have found a positive relationship between fat intake and adiposity in children even after controlling for confounding factors [41,42]. The main objection to the notion that dietary fat is responsible for the accelerated pediatric obesity epidemic is the fact that at the same time the prevalence of childhood obesity was increasing, the consumption of dietary fat in different populations was decreasing. Although fat eaten in excess leads to obesity, there is not strong enough evidence that fat intake is the chief reason for the ascending trend of childhood obesity.

#### Other dietary factors

there is a growing body of evidence suggesting that increasing dairy intake by about two servings per day could reduce the risk of overweight by up to 70% [43]. In addition, calcium intake was associated with 21% reduced risk of development of insulin resistance among overweight younger adults and may reduce diabetes risk [44]. Higher calcium intake and more dairy servings per day were associated with reduced adiposity in children studied longitudinally [45,46]. There are few data reporting the relation between calcium or dairy intake and obesity among children.

Between 1970 and 1997, the United State Department of Agriculture (USDA) surveys indicated an increase of 118% of per capita consumption of carbonated drinks, and a decline of 23% for beverage milk [47]. Soft drink intake has been associated with the epidemic of obesity [48] and type II diabetes [49] among children. While it is possible that drinking soda instead of milk would result in higher intake of total energy, it cannot be concluded definitively that sugar containing soft drinks promote weight gain because they displace dairy products.

### II. Physical Activity

It has been hypothesized that a steady decline in physical activity among all age groups has heavily contributed to rising rates of obesity all around the world. Physical activity strongly influenced weight gain in a study of monozygotic twins [50]. Numerous studies have shown that sedentary behaviors like watching television and playing computer games are associated with increased prevalence of obesity [51,52]. Furthermore, parents report that they prefer having their children watch television at home rather than play outside unattended because parents are then able to complete their chores while keeping an eye on their children [53]. In addition, increased proportions of children who are being driven to school and low participation rates in sports and physical education, particularly among adolescent girls [51], are also associated with increased obesity prevalence. Since both parental and children's choices fashion these behaviors, it is not surprising that overweight children tend to have overweight parents and are themselves more likely to grow into overweight adults than normal weight children [54]. In response to the significant impact that the cultural environment of a child has on his/her daily choices, promoting a more active lifestyle has wide ranging health benefits and minimal risk, making it a promising public health recommendation.

## Prevention

Almost all public health researchers and clinicians agree that prevention could be the key strategy for controlling the current epidemic of obesity [55]. Prevention may include primary prevention of overweight or obesity itself, secondary prevention or avoidance of weight regains following weight loss, and prevention of further weight increases in obese individuals unable to lose weight. Until now, most approaches have focused on changing the behavior of individuals on diet and exercise and it seems that these strategies have had little impact on the growing increase of the obesity epidemic.

### What age group is the priority for starting prevention?

Children are often considered the priority population for intervention strategies because, firstly, weight loss in adulthood is difficult and there are a greater number of potential interventions for children than for adults. Schools are a natural setting for influencing the food and physical activity environments of children. Other settings such as preschool institutions and after-school care services will have similar opportunities for action. Secondly, it is difficult to reduce excessive weight in adults once it becomes established. Therefore it would be more sensible to initiate prevention and treatment of obesity during childhood. Prevention may be achieved through a variety of interventions targeting built environment, physical activity and diet.

### Built Environment

The challenge ahead is to identify obesogenic environments and influence them so that healthier choices are more available, easier to access, and widely promoted to a large proportion of the community (Table 2). The neighborhood is a key setting that can be used for intervention. It encompasses the walking network (footpaths and trails, etc.), the cycling network (roads and cycle paths), public open spaces (parks) and recreation facilities (recreation centers, etc.). While increasing the amount of public open space might be difficult within an existing built environment, protecting the loss of such spaces requires strong support within the community. Although the local environment, both school and the wider community, plays an important role in shaping children's physical activity, the smaller scale of the home environment is also very important in relation to shaping children's eating behaviors and physical activity patterns. Surprisingly, we know very little about specific home influences and as a setting, it is difficult to influence because of the total numbers and heterogeneity of homes and the limited options for access [56]. Of all aspects of behavior in the home environment, however, television viewing has been researched in greatest detail [57-59].

**Table 2:** Some interventions strategies that could be considered for prevention of childhood obesity

I. Built environment
1. Walking network
a. Footpaths (designated safe walking path)
b. Trails (increasing safety in trails)
2. The cycling network
a. Roads (designated cycling routes)
b. Cycle paths
3. Public open spaces (parks)
4. Recreation facilities (providing safe and inexpensive recreation centers)

II. Physical activity
1. Increasing sports participation
2. Improving and increasing physical education time
3. Use school report cards to make the parents aware of their children's weight problem
4. Enhancing active modes of transport to and from school
a. Walking e.g. walking bus
b. Cycling
c. Public transport
III. TV watching
1. Restricting television viewing
2. Reducing eating in front of the television
3. Ban or restriction on television advertising to children

IV. Food sector
1. Applying a small tax on high-volume foods of low nutritional value (e.g. soft drinks, confectionery, and snack foods)
2. Food labeling and nutrition 'signposts' (e.g. logos for nutritious foods)
3. Implementing standards for product formulation

#### Physical activity

Stone et al. [60] reviewed the impact of 14 school-based interventions on physical activity knowledge and behavior. Most of the outcome variables showed significant improvements for the intervention. One interdisciplinary intervention program in the USA featured a curriculum-based approach to influence eating patterns, reduce sedentary behaviors (with a strong emphasis on television viewing), and promote higher activity levels among children of school grades 6 to 8. Evaluation at two years showed a reduction in obesity prevalence in girls (OR = 0.47; 95%CI: 0.24 – 0.93), but not in boys (OR = 0.85; 95%CI: 0.52 – 1.39) compared to controls. The reduction in television viewing (by approximately 30 min/day) was highly significant for both boys and girls. Increases in sports participation and/or physical education time would need policy-based changes at both school and education sector levels [61]. Similarly, increases in active modes of transport to and from school (walking, cycling, and public transport) would require policy changes at the school and local government levels, as well as support from parents and the community. In some communities a variety of such programs have been implemented e.g. road crossings, 'walking bus', and designated safe walking and cycling routes [51].

### Effects of dietary pattern and TV watching

It appears that gains can be made in obesity prevention through restricting television viewing. Although, it seems that reduced eating in front of the television is at least as important as increasing activity [58]. Fast foods are one of the most advertised products on television and children are often the targeted market. Reducing the huge volume of marketing of energy-dense foods and drinks and fast-food restaurants to young children, particularly through the powerful media of television, is a potential strategy that has been advocated. Television advertising to children under 12 years of age has not been permitted in Sweden since commercial television began over a decade ago, although children's television programs from other countries, and through satellite television, probably dilute the impact of the ban in Sweden. Norway, Denmark, Austria, Ireland, Australia, and Greece also have some restrictions on television advertising to young children [51]. The fact that children would still be seeing some television advertisements during adult programs or other types of marketing, such as billboards, does not contradict the rationale for the control on the television watching of young children.

### Food Sector

Food prices have a marked influence on food-buying behaviour and, consequently, on nutrient intake [62]. A small tax (but large enough to affect sales) on high-volume foods of low nutritional value, such as soft drinks, confectionery, and snack foods, may discourage their use. Such taxes currently applied in some parts of the USA and Canada [63]. In addition, food labeling and nutrition 'signposts' such as logos that indicate that a food meets certain nutrition standards might help consumers make choices of healthy foods. An example is the 'Pick the Tick' symbol program run by the National Heart Foundations in Australia and New Zealand [64]. The 'Pick the Tick' symbols made it easier for consumers to identify healthier food choices and are frequently used by shoppers. In addition, the nutrition criteria for the products serve as 'de facto' standards for product formulation, and many manufacturers will formulate or reformulate products to meet those standards.

### Effectiveness of the prevention methods

It has been shown that focusing on reducing sedentary behaviour and encouraging free play has been more effective than focusing on forced exercise or reducing food intake in preventing already obese children from gaining more weight [65]. Recent efforts in preventing obesity include the initiative of using school report cards to make the parents aware of their children's weight problem. Health report cards are believed to aid prevention of obesity. In a study in the Boston area, parents who received health and fitness report cards were almost twice as likely to know or acknowledge that their child was actually overweight than those parents who did not get a report card [66]. They also were over twice as likely to plan weight-control activities for their overweight children.

A summary of prevention and intervention strategies is presented in Table 2.

## Conclusion

Obesity is a chronic disorder that has multiple causes. Overweight and obesity in childhood have significant impact on both physical and psychological health. In addition, psychological disorders such as depression occur with increased frequency in obese children. Overweight children are more likely to have cardiovascular and digestive diseases in adulthood as compared with those who are lean. It is believed that both over-consumption of calories and reduced physical activity are mainly involved in childhood obesity.

Apparently, primary or secondary prevention could be the key plan for controlling the current epidemic of obesity and these strategies seem to be more effective in children than in adults. A number of potential effective plans can be implemented to target built environment, physical activity, and diet. These strategies can be initiated at home and in preschool institutions, schools or after-school care services as natural setting for influencing the diet and physical activity and at home and work for adults. Both groups can benefit from an appropriate built environment. However, further research needs to examine the most effective strategies of intervention, prevention, and treatment of obesity. These strategies should be culture specific, ethnical, and consider the socio-economical aspects of the targeting population.

## Abbreviations

NHANES National Health and Nutrition Examination Survey

MONICA Multinational Monitoring of trends and determinants in cardiovascular disease

BF Body Fat

BMI Body Mass Index

BIA Bioelectrical Impedance Analysis

MRI Magnetic Resonance Imaging

TBW Total Body Water

FFM Fat-Free Mass

USDA United State Department of Agriculture

## Authors' contributions

All authors had equal contribution in writing this manuscript.
